# Hemodynamic and analgesic effect of intrathecal fentanyl with bupivacaine in patients undergoing elective cesarean section; a prospective cohort study

**DOI:** 10.1371/journal.pone.0268318

**Published:** 2022-07-07

**Authors:** Ayub Mohammed Ebrie, Misrak Woldeyohanis, Bedru Jemal Abafita, Siraj Ahmed Ali, Abebayehu Zemedkun, Yusuf Yimer, Zewetir Ashebir, Salih Mohammed

**Affiliations:** 1 Lecturer Department of Anesthesiology, Dilla University College of Medicine and Health Sciences, Dilla, Ethiopia; 2 Lecturer Department of Anesthesia, Addis Ababa University College of Health Sciences, Addis Ababa, Ethiopia; 3 Lecturer, Department of Anesthesiology, Wollo University, College of Health Sciences and Medicine, Dessie, Ethiopia; Stanford University School of Medicine, UNITED STATES

## Abstract

**Background:**

Spinal anesthesia with bupivacaine has side effects such as hypotension, respiratory depression, vomiting, and shivering. The side effects are dose-dependent, therefore different approaches have been attempted to avoid spinal-induced complications including lowering the dose of local anesthetic and mixing it with additives like Neuraxial opioids.

**Objective:**

To compare the Hemodynamic and analgesic effects of intrathecal fentanyl as an adjuvant with low and conventional doses of bupivacaine in patients undergoing elective cesarean section under spinal anesthesia.

**Methodology:**

An institutional-based prospective cohort study was conducted on 90 patients. Data was collected with chart review, intraoperative observation, and postoperatively patient interview. Data was entered into EPI INFO and transport to SPSS version 23 for analysis of variables using one-way ANOVA, Kruskal Wallis H rank test, and chi-square.

**Result:**

Hypotension but not bradycardia, was significantly frequent in a conventional dose of bupivacaine alone (CB) group and a conventional dose of bupivacaine with fentanyl (CBF) groups than that of the lower dose of bupivacaine with fentanyl (LBF) groups. Duration of analgesia was significantly longer in LBF (248± 35.6 minutes) and in CBF groups (260.3±40.3 minutes) than in CB group (167.10 ± 31.45 minutes). Time for the first analgesic request was significantly later in LBF (304±47.8 minutes) and CBF (294.6±99.5 minutes) groups than that in CB group (177±25.88 minutes).

**Conclusion:**

The Lower dose of bupivacaine is associated with less risk of hypotension and faster recovery. Adding fentanyl with the lower dose of bupivacaine in spinal anesthesia for cesarean section could provide comparable anesthesia with the lower risk of hypotension and longer postoperative analgesia.

## 1. Introduction

Cesarean section (CS) is an operative technique by which a fetus is delivered through an abdominal and uterine incision. [1]. It is one of the most common surgeries in the world, which has been increased in the last 21 years [2]. The 2016 EDHS program found that the rate of cesarean delivery in Ethiopia is around 2% even if it varies across the seven administrative regions of Ethiopia, of all Addis Ababa had the highest rate of cesarean section which accounts for around 21.4% [3].

Spinal anesthesia has become the anesthetic technique of choice for CS and has resulted in a reduction in maternal mortality [4, 5]. Simple technique, fast in its effect, and uniform sensory and motor blocks are the advantages of spinal anesthesia; however post-spinal hypotension is common in women who receive spinal anesthesia for cesarean delivery, with an incidence of up to 58.4% [6]. Spinal hypotension is commonly associated with maternal nausea and vomiting, reduces uteroplacental blood flow, which may cause fetal acidosis, particularly in situations in which there is already a fetal compromise and it may be an important contributory factor for maternal death related to regional anesthesia [7].

Postoperative pain after CS is reported to be higher (an unpleasant outcome for women and may result in delayed ambulation, prolonged time for discharge from hospital, poor bonding with the newborn, low satisfaction scores, and delay breastfeeding [8]. In contrast, effective analgesia may permit improved mother-child bonding, early ambulation and discharge, greater patient satisfaction, and early breastfeeding [9]. Neuraxial administration of opioids along with local anesthetics improves the quality of intraoperative analgesia and provides postoperative pain relief for a longer duration than local anesthetics alone [10, 11].

Bupivacaine is the most commonly used drug worldwide for spinal anesthesia in CS. Spinal anesthesia with local anesthetic agents, especially bupivacaine, has side effects such as hypotension, respiratory depression, vomiting, and shivering in a dose-dependent fashion. Its side effects are dose-dependent, therefore different approaches have been attempted including the use of a small dose of bupivacaine or by lowering the dose of local anesthetic and mixing it with additives like Neuraxial opioids [12, 13].

The spinal anesthesia in a cesarean section is still presenting a challenge to the anesthetist in the form of either severe hypotension from a large bupivacaine dose or insufficient satisfactory anesthesia level conditions as a result of a small bupivacaine dose [14].

A recent systemic review and meta-analysis of Ten Randomized trials show Incidence of hypotension was less likely in mothers who received low dose bupivacaine with Fentanyl as compared to those with conventional dose of bupivacaine alone (RR = 0.43, 95% confidence interval (CI) 0.12–0.47, ten trials, 552 participants). The review revealed that Low dose bupivacaine combined with intrathecal Fentanyl decrease the incidence of hypotension [15].

Among synthetic opioids, fentanyl is favorable due to greater potency, faster onset of action, and rapid redistribution with an associated decrease in the plasma concentration of the drug and thus enhancing the early postoperative analgesia [16].

This study can be used as a source of information for further researchers and a sole input to the literature. In addition to this, the result of this study will be helpful for program planners and policymakers to devise different strategies that help to improve and select an appropriate combination of bupivacaine and fentanyl to increase maternal satisfaction with spinal anesthesia. This study aimed to compare analgesic effectiveness and hemodynamic change of intrathecal fentanyl as an adjuvant with a low dose and conventional dose of bupivacaine in parturients undergoing elective cesarean section under spinal anesthesia.

## 2. Methods and materials

An institutional-based prospective cohort study was conducted from December 1, 2018, to march 30, 2019 in Mahatma Gandhi Memorial Hospital, one of the public hospitals in Addis Ababa, the capital of Ethiopia. Ethical clearance was obtained from the institutional review board of Addis Ababa University College of health science department of anesthesia with a protocol number of **27/18/Anes** before the start of the study. An official support letter was written to the Hospital and permission for data collection was sought from the responsible authorities. Verbal as well as written informed consent was obtained from each participant. All parturients who underwent elective cesarean delivery under spinal anesthesia were included in this study. Parturients with preexisting hypertension or PIH requiring treatment, history of chronic renal, cardiac, or liver disease, Patients with extreme height (<140 cm and >175 cm), those who underwent spinal anesthesia with added adjuvant like trans abdominal plane block(TAP block) and adjuvants other than intrathecal fentanyl, Those having the volume of surgical bleeding more than 1500 cc were excluded from the study. The protocol and routine procedures before, during, and after CS at the study site were as follows:

On arrival of the patients to the operative theater, and after application of the routine hospital monitoring protocol, HR, noninvasive blood pressure, and SPO2 has been recorded before the institution of spinal anesthesia, then all patients received spinal anesthesia with 2–3 ml of 0.5% bupivacaine alone or with different types of adjuvants like fentanyl with a dose of either 15 or 25 mcg based on the responsible anesthetist’s preference. So that we included to our study those taking 8 mg of bupivacaine with 25 mcg fentanyl or 10 mg bupivacaine with 25 mcg fentanyl or 10 mg bupivacaine alone. Patients were not informed about which drug to administer for them.

After this, all patients were repositioned in a supine position and the level of sensory and degree of motor block was assessed. During each procedure, the data collectors observed the intraoperative procedures and those patients fulfilling the inclusion criteria included in our study, and then intraoperative data were recorded. After SA is performed the data collectors recorded the maximum sensory block assessed with the patient’s ability to distinguish the sharpness created by the tip of the needle(pinprick method) and motor block level which is routinely examined by examining skeletal muscle strength criteria using modified Bromage scale. Any need for supplemental analgesia, conversion to general anesthesia, and other intraoperative complications were recorded by the data collector. The neonates’ Apgar score had also been recorded.

We the investigators did not participate in the perioperative patient management and post-operative pain management option.

Post-operatively each patient was interviewed and their chart was also reviewed. Group LBF are those laboring mothers taking 25 mcg of intrathecal fentanyl with 0.5% of 8 mg bupivacaine, group CBF is those who take 25 mcg of intrathecal fentanyl with 0.5% of 10 mg bupivacaine and group CB are those taking 0.5% of 10 mg of bupivacaine only. The fentanyl dose of 25mcg is chosen based on the recommendations in the review of Hamber and Viscomi [17]. One of the data collectors takes and observes intraoperative necessary information.

Starting from the immediate postoperative time, presence, and scale of pain, time for the first analgesic request as well as analgesics need was assessed by the other trained data collector. This assessment was done at 1hr, 2 hrs, 3hrs, 4hrs, 5hrs, and 6hrs for VAS score. All patients were also assessed within 12 hrs after surgery for potential drug complications such as nausea and vomiting, itching, etc and the neonatal Apgar score was also assessed in the first and fifth minute of delivery.

The categories of patients were identified by the data collectors. The VAS was determined by the patient marking their pain intensity on a line 10 cm long. Two BSC anesthetists were selected to collect data and one-day training was given on how to collect data. Another MSC anesthetist was assigned to assist and supervise data collectors.

**Hemodynamic**: in this research tends to refer to arterial blood pressure.
**Hypotension**: defined as a systolic blood pressure of below 90mmhg or lower than 30% of starting systolic blood pressure or MAP less than 70mmhg [7].
**Time for first analgesic request**: is the time from intrathecal injection to first request for analgesia [18].
**Duration of analgesia**: is considered as time duration from spinal injection to the time when a patient had discomfort or pain.
**Bromage scale**: is a criterion used for examining skeletal muscle strength which is rated as 0 = no paralysis, 1 = only able to move the knee, 2 = only able to move feet, 3 = inability to move the leg or knee [19].
**Low dose of Bupivacaine**: is considered as a dose of intrathecal bupivacaine of <8 mg for cesarean section [20].
**Conventional dose of bupivacaine**: Is considered when its intrathecal dose is >8 mg for cesarean section [20].

### Sample size and sampling technique

The primary endpoint of our study was to compare hemodynamic status and analgesic effect so that sample size estimation based on the largest sample size were used and calculated by using a priori power analysis (G Power version 3.01) based on the results of a pilot study done at Gandhi memorial hospital on 15 patients (5 for each group) and taking mean systolic blood pressure measurement in mmHg at 10 minute which is 99.6,90 and 94 for LBF, CBF and CB group respectively. The common pooled standard deviation was 9.6 for all three groups. Controlling for the probability of a Type I error at alpha = 0.017 (the alpha level was reduced using a Bonferroni correction, 0.05/3 = 0.017, to allow for comparisons of all three groups), and a power of 80% was used. The calculated sample size was 81; by adding a 10%, attrition rate, and assuming a balanced design the total sample size was 90. Based on situational analysis of Gandi memorial hospital surgical log book shows 360 patients estimated to undergo cesarean section within the hospital during the study period, So 90 participants were recruited by systematic sampling technique with the probability of about 25%. k = N/n = 4. according to the study, hospital routine protocol daily operated case lists are posted in the operative room board so that the first study participant was selected by lottery method manually and the other participants based on k = 4. If the selected patient didn’t take the dose that we want we wait for the next one and if so included it in our study.

### Data processing and analysis

Data were entered into Epi-info 7 and exported to SPSS V 23 for analysis. The data were tested for normality using histogram and Shapiro–Wilk normality test (p-value >0.05 taken as normally distributed) and homogeneity of variance was tested by Levene’s test (p-value >0.05). Normally distributed and continuous data were analyzed using one-way analysis of variance (ANOVA) with post hoc analysis for multiple tests and non-normally distributed data were analyzed using the kuruska-Wallis H rank test. The comparisons of categorical variables were analyzed using the Pearson chi-square test. Data were presented as mean ±SD for normally distributed, median ± IQR for non-normally distributed, and categorical data were presented as numbers and frequencies (percentages). P-values <0.05 were considered statistically significant.

## 3. RESULT

### 3.1 Socio-demographic characteristics of the participants

Ninety pregnant mothers who underwent cesarean delivery under spinal anesthesia during the study period were included. Of these patients, 30 mothers were given intrathecal fentanyl (25mcg) with (8 mg) 0.5% bupivacaine (LBF group), 30 patients fentanyl 25 mcg with (10mg) (0.5%) bupivacaine (CBF group) and 30 patients 10 mg 0.5% bupivacaine alone (CB group). There was no statistically significant difference among the groups with socio-demographic data and also there is no significant difference in the total amount of preloaded fluid, the total amount of estimated blood loss, and the total amount of fluid administered intraoperatively with a p-value of >0.05 as shown in [Table 1].

**Table 1:** Demographic characteristics of patients undergoing elective cesarean section with differet doses and adjuvamts of spinal anesthesia.

		LBF-group(n = 30)	CBF-group(n = 30)	CB-group(n = 30)	P-value
Age in years		27(6)	26(4)	27(7)	0.530
Height in meter		1.59 ±0.27	1.60±0.28	1.60±1.03	0.394
Weight in kg		60.8±3.4	62.9±7.3	61.3±4.4	0.292
BMI		23.7±0.97	24.3±2.8	23.8±1.8	0.408
Parity	**I**	10(33.3%)	8(26.7%)	6(20%)	0.099
	**>I**	20(66.6%)	22(73.3%)	24(80%)	
Gestational age in weeks		39.0±1.24	39.3±1,26	39.8±1.76	0.12
Amount of fluid preloaded		540±223.7	550±241.7	603±188	0.552
The total amount of fluid given Intra op in ml		1570±475	1413±506	1430±521	0.417
Total surgical blood loss		500(200)	400(150)	500(250)	0.114

Hint: Value are presented as Mean±SD: One way ANOVA test, Median (IQR): Kuruska-Wallis H rank test, Number(%): chi-square test and p<0.05 is statistically significant.

### 3.2 Comparisons of hemodynamic parameters among groups

Systolic and diastolic blood pressures were decreased significantly(P < 0.05) in the first 30 minutes of spinal anesthesia in the conventional dose of bupivacaine groups even with and without the addition of fentanyl when compared to the low dose bupivacaine with the fentanyl group.

**Fig 1:**
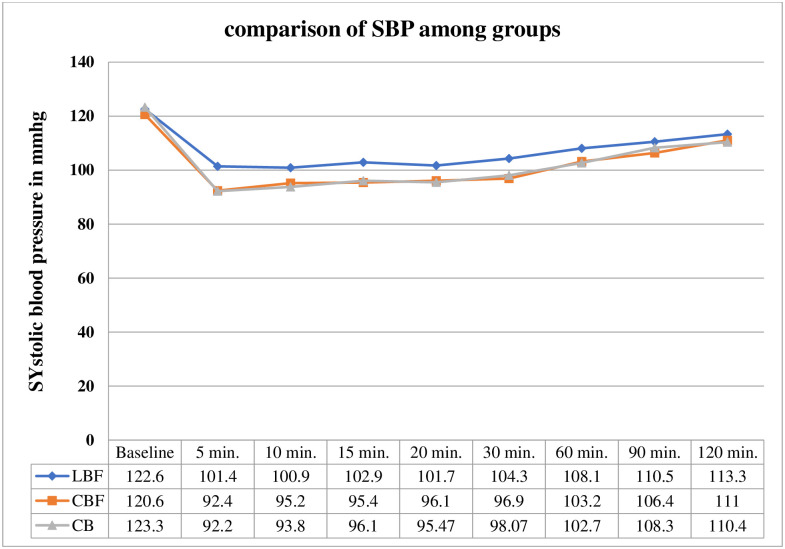
Line graph shows Systolic blood pressure (mmHg) measurement at different time intervals of patients underwent elective cesarean section under spinal anesthesia at gandhi memorial hospital addis ababa, Ethiopia 2019.

The peak decrease in systolic blood pressure was recorded in the first 10 to 15 minutes in all three groups. There is no statistically significant difference in systolic blood pressure between CBF and CB group at all-time intervals as shown in with p-value > 0.05 [Fig 1].

There was no statistically significant difference in mean heart rate at various time intervals among groups as shown in [Table 2].

**Table 2:** Heart rate measurement of patients undergoing elective cesarean section with different doses and adjuvants of spinal anesthesia.

		Baseline	5 min	10 min	20 min	30 min	60 min	90 min	120 min
LBF-group	Mean±SD	90.0±3.8	82.23±6.87	82.8±7	87.8±8.3	85.2±8.6	85.6±10.7	86.8±6.4	87.07±6.7
CBF- group	Mean±SD	92.9±4.3	78±8.5	84.5±9.4	86.3±10.2	84.3±7.7	87.6±9.0	88.2±9.4	88.1±6.9
CB-group	Mean±SD	91.6±5.3	82.1±9.4	81.4±7.8	85.7±10.8	83.8±8.8	87.9±8.6	87.2±7.6	87.9±7.0
P-value		0.57	0.086	0.343	0.693	0.494	0.589	0.776	0.814

Hint: P-value <0.05 is taken as significant

### 3.3 Comparisons of Characteristics of spinal anesthesia among groups

The time required for the onset of the target sensory block of the T6 dermatome was significantly faster in group LBF and in group than in group CB. However, there was no significant difference between both the groups in the quality of surgical anesthesia.

The duration of postoperative analgesia was significantly prolonged in the LBF group and the CBF group than in the CBF group. In post hoc analysis there was no significant difference in postoperative analgesia duration. The quality of the sensory blockade was good referring to no requirement of any analgesic support intraoperatively in all three groups. Quality of motor blockade assessed by Bromage scale showed adequate muscle relaxation in all groups with 90% of patients had level **IV** motor block and the other 10% had level **III** motor block with no significant difference among groups (P-value <0.05) (Table 3).

**Table 3:** Characteristic of spinal anesthesia and duration of analgesia of mother’s undergone elective cesarean section.

	LBF(n = 30)	CBF(n = 30)	CB(n = 30)	P-value
Highest sensory level (dermatome)	T4	T4	T4	-
Time of Onset of sensory level T6 (minute)	3.13±0.57	3.07±0.69	4.03±0.99	0.000
Duration of grade 0 motor block	120(30)	150(50)	150(20	0.002
Duration of analgesia (minute)	248± 35.6	260.3±40.3	167.10 ± 31.45	0.001
Time for first analgesic request	304±47.8	294.6±99.5	177±25.88	0.001

Values are presented as Mean±SD: One way ANOVA test, Median (IQR): Kuruska-Wallis H rank, *P-*value <0.05 taken as significant

### 3.4 Comparing Postoperative time to first analgesic request among groups

**Fig 2:**
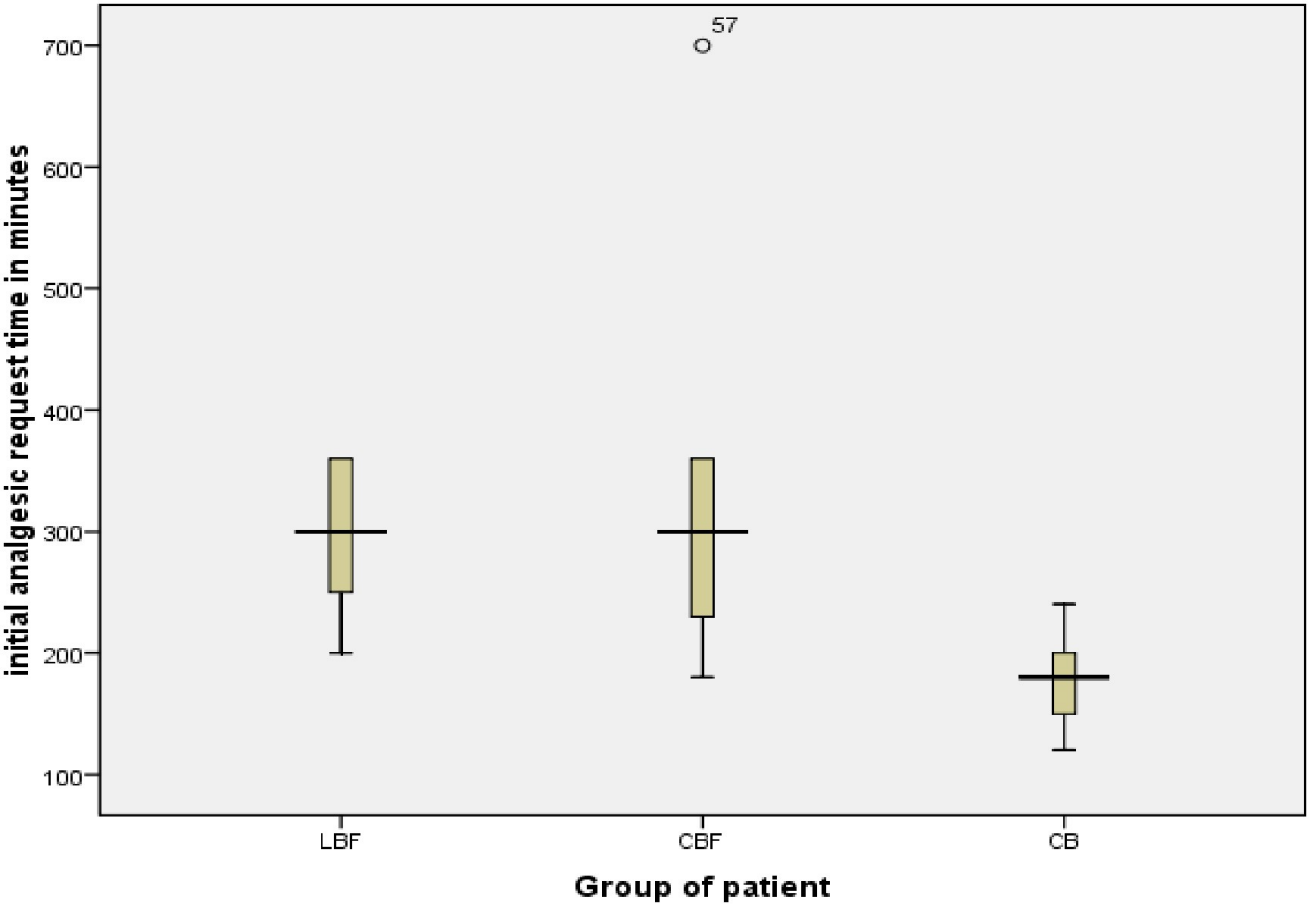
Comparison of meantime for postoperative first analgesic request among groups Of patients undergoing elective cesarean section with different doses and adjuvants of spinal anesthesia.

Postoperatively, a one-way ANOVA test showed that the time from intrathecal injection to the first analgesic request was significantly different among the three groups (*P* <0.05) [Fig 2]. In post hoc multiple test analysis there was no significant difference in time for first analgesic request between LBF and CBF groups with a p-value of 0.889.

### 3.5 Comparison of Incidence of perioperative complications among groups

**Fig 3:**
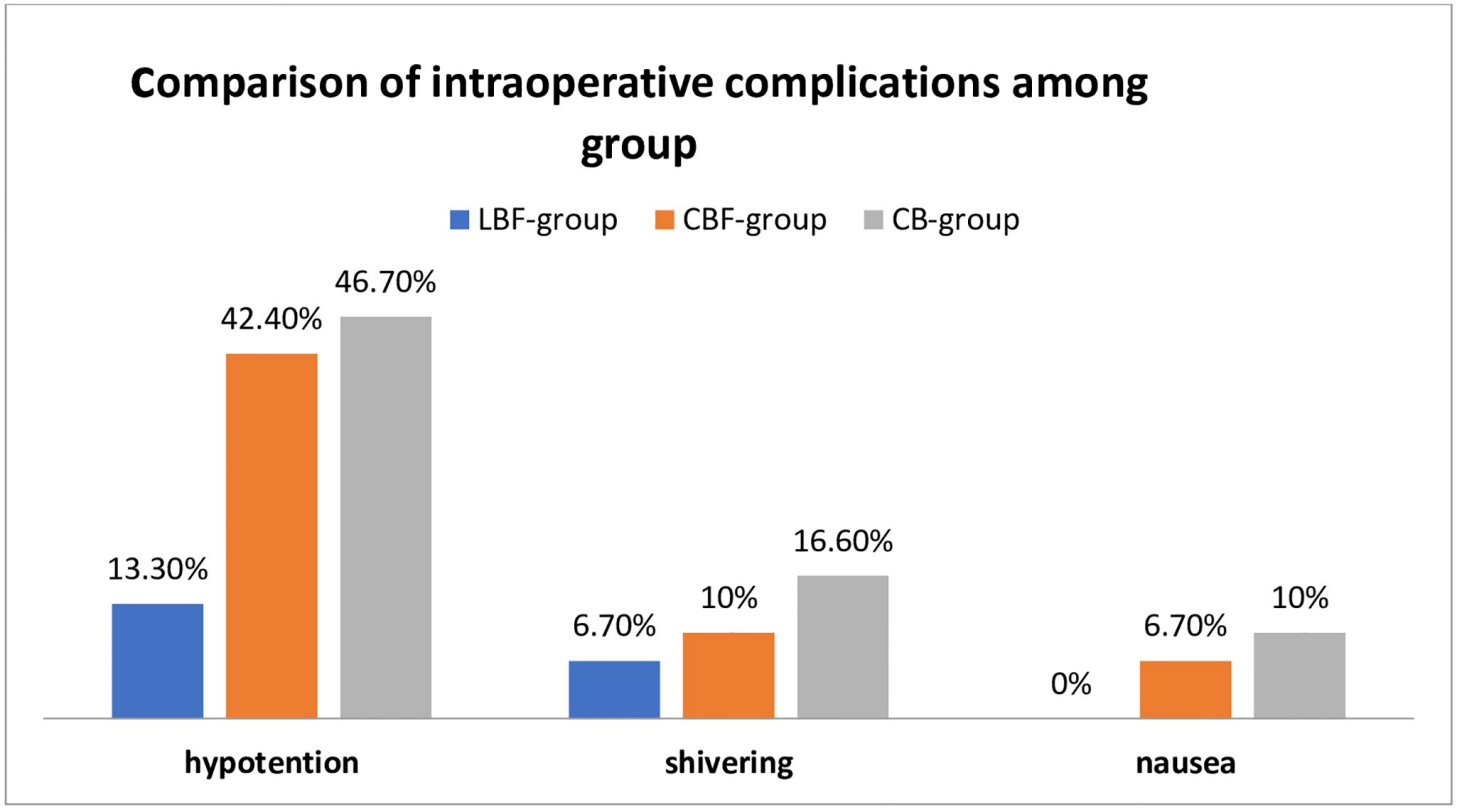
A bar graph representing the commonest intraoperative complications encountered among groups of patients undergoing elective cesarean section with different doses and adjuvants of spinal anesthesia.

There was statistically significant difference in incidence of hypotension which is higher in CBF (42.2%) and CB (46.7%) groups when compared with the LBF group (13.3%). Hypotension was treated with IV fluid and vaso- active drugs were not administered for any of them. Other complications such as nausea, vomiting, and pruritus were mild and further treatment was not needed. None of the patients develop respiratory depression and the first and five-minute neonatal Apgar score was between 7 and 10 in all groups. 2 (6.7%) patients in the CBF group and 3(10%) patients in the CB group developed nausea but none of the patients in the LBF group developed nausea. None of the patients developed vomiting in LBF and CBF group but 2(6.7%) of patients experienced vomiting from the CB group which is not statistically significant. 2 (6.7%) of patients developed shivering in LBF, 1(3.3%) in CBF group and 5(16.6%) in CB group with a p-value of 0.005. (Fig 3).

## 4. Discussion

Systolic and diastolic blood pressures were decreased significantly(P < 0.05) in the first 30 minutes of spinal anesthesia in the conventional dose of bupivacaine groups even with and without the addition of fentanyl when compared to the low dose bupivacaine with the fentanyl group. A similar finding was also observed by Himabindu G. et al, that compares hemodynamic stability and analgesic effect of 7.5 mg bupivacaine and 10 mg bupivacaine alone. Bogra et al. [13], Seyedhejazi and Madarek [21] also show similar findings to our study. This could be due to more sympathetic blockade by higher doses of bupivacaine in the conventional doses of bupivacaine groups.

In a post-hoc analysis, there is no significant difference (p>0.05) in systolic blood pressure between CBF group and CB group in all time intervals. This shows hypotension was associated with higher bupivacaine dosage and the addition of fentanyl had no significant effect on maternal hemodynamics. Our study is supported by a study done in Ethiopia by kassaw et al. found that no significant change in arterial blood pressure between the study group (10 mg bupivacaine and 25 mcg fentanyl) and the control group (12.5 mg bupivacaine alone) [22].

Regarding our study, there was a statistically significant difference in the incidence of hypotension which is higher in CBF (42.2%) and CB (46.7%) groups when compared with the LBF group (13.3%). Mhamed S. et al. also showed similar findings as 10 mg (group A) and 7.5 mg (group B) isobaric bupivacaine both with 25 μg of fentanyl and 100 μg of morphine in spinal anesthesia for cesarean section showed the incidence of low blood pressure was markedly higher in the group A than in group B (88% vs 68%p = 0.03) [23].

In our study, no patient needs vasopressor treatment for hypotension. In the previous study by Mhammed et al. a larger dose of ephedrine was needed in the bupivacaine alone group than fentanyl treated groups (32±23 vs 19±16 mg; p = 0.004) respectively [23]. The reason why our patients do not need vasopressor might be due to the protocol difference in managing spinal hypotension.

According to our study, mothers in the LBF group and the CBF group had a significantly long time for the first analgesic request compared to mothers in the CB group, (304± 47.8), (294.6 ± 99.5) and (177±25.88) in minutes respectively. In contour to our study Biswas BN et al. showed that analgesia request time was shorter in the bupivacaine alone group (150±10.48 minute) compared to the fentanyl treated group (248 ± 11.7 minutes) respectively [24].

Our study showed that the addition of 25 mcg fentanyl increased the total duration of complete analgesia which was 248± 35.6 in minutes in the LBF group,260.3±40.3 minutes in the CBF group compared to bupivacaine alone group which is 167.10 ± 31.45 in minutes with p-value = 0.001. our study is in line with a study done by Shashikala TK et al. grouped 99 parturients into, FB group who had been given 2ml of 0.5% bupivacaine with 12.5mcg fentanyl and the BC group receiving 2ml 0.5% bupivacaine only [25]. They found that the total duration of meantime analgesia between Group BC and Group FB was 165 ± 29.8 minutes in Group BC and 259.4 ± 35.3 minutes in Group FB respectively.

In our study, there was no statistically significant difference in mean heart rate at various time intervals in all groups, which was a similar finding with the previous study by Shashikala TK et al. and Jaishri et al. [13, 25]. However, a study in Nepal showed that the incidence of bradycardia was 5.7% in the control group and 2.8% in Group BF with no significant variation in the group [26]. In comparison to the above two studies, there were no cases of bradycardia in our patients. This might be due to the relatively lower dose of bupivacaine that we used when compared with the above studies, as proven scientifically the larger the bupivacaine dose the higher the height of the block will result so that it will block cardio-accelerator fibers above T4 and causes bradycardia.

Concerning our study, 2 (6.7%) patients in the CBF group and 3(10%) patients in the CB group developed nausea but none of the patients in the LBF group developed nausea. none of the patients developed vomiting in LBF and CBF group but 2(6.7%) of patients experienced vomiting from the CB group which is not statistically significant. A study from Iran found that adding 25mcg fentanyl reduced the incidence of nausea (7 patients) and vomiting (4 patients) in Bupivacaine fentanyl group compared with 15 patient developed nausea and 12 patients developed vomiting in their Bupivacaine alone group [2]. Our study is comparable with these results and our possible explanation for the reduction of nausea/vomiting in fentanyl added groups might be due to an increased dose of fentanyl (25mcg), a better quality of analgesia, and good hemodynamic stability in the LBF group.

In the group-LBF, one patient complained of mild pruritus and none of the patients developed pruritus in CBF and CB groups, these might be because of side effects of fentanyl, even the etiology of it was not ascertained, The possible explanation is likely due to the cephalic migration of the opioids in CSF and its subsequent interaction with opioid receptors in the trigeminal nucleus. Similar findings were observed in the previous studies mentioned above by Himabindu G. et al. and Cowan et al. [27, 28]. The study by Jaishri et al. [13] also observed no incidence of pruritus in fentanyl-treated groups. Another study in India using 12.5 mcg fentanyl with 10mg bupivacaine compared with 10 mg bupivacaine, they found that no patients had pruritus in either of the group [19].

In our study 2 (6.7%) of patients developed shivering in LBF, 1(3.3%) in CBF group and 5(16.6%) in CB group with a p-value of 0.005 which correlate with the study by Jaishri et al. shows a lower rate of shivering in fentanyl treated groups [13].

## Conclusion and recommendation

Adding fentanyl with the lower dose of bupivacaine in spinal anesthesia for cesarean section could provide comparable anesthesia with a lower risk of hypotension and longer postoperative analgesia. We recommend the use of 25mcg intrathecal fentanyl with 8mg bupivacaine as appropriate to improve the hemodynamic status of patients, in addition, to improve postoperative analgesia for cesarean section.

## Supporting information

S1 Data Hemodynamic and analgesic effect of intrathecal fentanyl with bupivacaine data. (SAV)

## Abbreviations

ASA: American Society of Anesthesiologists
CB: conventional dose of bupivacaine alone
CBF: conventional dose bupivacaine with fentanyl
DBP: Diastolic blood pressure
GA: General anesthesia
LBF: low dose of bupivacaine with fentanyl
MAP: Mean arterial blood pressure
PI: Principal Investigator
PIH: Pregnancy Induced Hypertension
SA: Spinal anesthesia
SBP: Systolic blood pressure
VAS: Visual Analogue Score
